# Insects as disease vectors: Historical and contemporary analysis of entomological warfare

**DOI:** 10.1007/s13280-025-02212-x

**Published:** 2025-07-19

**Authors:** Abdelwahab Khalil, Shaimaa Abdelgawad, Aya A. El-Hareef, Aliaa A. Maatouk, Lamiaa M. Genaidy, Nour Y. S. Yassin, Abeer M. Salem

**Affiliations:** 1 Entomology Division, Zoology Department, Faculty of Science, Beni-Suef University, Beni-Suef, 62521 Egypt; 2 Zoology Department, Faculty of Science, Beni-Suef University, Beni-Suef, 62521 Egypt; 3 Physiology Division, Department of Zoology, Faculty of Science, Beni-Suef University, Beni-Suef, 62521 Egypt; 4 Department of Entomology, Faculty of Science, Cairo University, P.O. Box 12613, Giza, Egypt; 5 Department of Biotechnology, Faculty of Science, Cairo University, P.O. Box 12613, Giza, Egypt

**Keywords:** Ancient civilizations, Bioweapon, Entomological warfare, Entomological weapon, Insect based warfare

## Abstract

Entomological warfare has been known since ancient civilizations used insects to spread disease among enemies. Advances in science and technology have enhanced the potential for using insects as vectors for viruses and toxins in warfare, including both traditional methods and new innovations like genetic engineering. This review aims to: (i) explore the historical and contemporary roles of insects in biological warfare; (ii) examine the ethical, legal, and ecological challenges associated with this form of warfare; and (iii) outline historical uses of insects as weapons, from releasing disease-carrying mosquitoes to creating genetically modified insects. It highlights the complexities of legal and ethical frameworks governing entomological warfare and potential environmental impacts. While insects offer unique advantages as biological agents, their use in combat raises significant ethical and environmental concerns. Therefore, there is an urgent need for international cooperation and regulatory oversight to ensure responsible and ethical applications of entomological warfare technologies.

## Background

Throughout history, humans have recognized the potential of insects as agents of biological warfare, exploiting their innate capabilities to inflict harm upon adversaries (Majumdar 2019). From ancient civilizations employing beehives as weapons to modern scientific advancements in genetic engineering, the use of insects in warfare has evolved and diversified (Rather et al. 2020). Historically, insects have played pivotal roles in conflicts dating back to antiquity. Ancient civilizations, such as the Greeks, Romans, and Chinese, leveraged insects like bees and hornets to disrupt enemy forces. Bees were deployed to instill fear and confusion among adversaries, while hornets were unleashed to induce chaos and inflict physical harm. These early examples demonstrate humanity’s early recognition of insects’ potential as weapons of war. In the modern era, the understanding of entomological warfare has expanded with advancements in science and technology (Mirghaed et al. 2022). During World War II (WWII**)**, both Axis and Allied powers explored the use of insects as vectors for disease transmission. Notably, the Japanese Imperial Army researched weaponizing fleas infected with plague, resulting in devastating outbreaks in Chinese cities (Wilson and Daniel 2019).

Similarly, Allied forces investigated the possibility of using mosquitoes to spread malaria among enemy troops, although these efforts were largely experimental and only partially implemented (Howie-Willis 2016). Contemporary scientific developments have further expanded the scope of entomological warfare, with researchers exploring genetic modification techniques to enhance insects’ capabilities as vectors of disease or carriers of toxins. The emergence of gene editing technologies like CRISPR-Cas9 has raised concerns about the potential misuse of such methods to engineer insects for malevolent purposes. Additionally, the advent of unmanned aerial vehicles (UAVs) equipped with insect-sized drones could facilitate the targeted delivery of insect-based weapons, posing new challenges for defense and security (Piergentili et al. 2021). Despite the potential strategic advantages entomological warfare may offer as targeted delivery and significant impacts on enemy forces, it also raises profound ethical questions and environmental risks. The deliberate release of disease-carrying insects could have catastrophic consequences, leading to uncontrollable epidemics and widespread suffering among civilian populations. Moreover, the use of insects in warfare blurs the distinction between combatants and non-combatants, challenging fundamental principles of humanitarian law and morality (Du Plessis 2017). This study presents a balanced perspective that recognizes these strategic advantages while highlighting the associated ethical and environmental concerns. Previous studies have typically concentrated on the historical dimensions of entomological warfare or on specific case studies of insect-borne disease outbreaks. In contrast, our review expands on this foundation by offering a thorough analysis that combines historical narratives with modern advancements in biotechnology and delivery methods. This integration fosters a deeper understanding of the evolving challenges and threats associated with entomological warfare. The present review aims to analyze the threat of the use of insects as weapons and the response capacities of the countries through the following steps: Provide a brief history of the biological weapon, focusing on entomological warfare, describe the benefits of insects as delivery devices for biological agents, and discuss the advantages they provide over traditional, mechanical delivery devices, and discuss contemporary threats and highlight recent advances in biotechnology that exacerbate the threat of biological weapons today.

## History of entomological warfare

Since ancient times, insects have been employed as biological weapons. Fleas were employed to disseminate the infection against the Asian Minor Kaffa City in the fourteenth century (Frith 2012). Colorado beetles were used by the Germans in WWII to destroy enemy crops (Lockwood 2008). Japanese also employed pestilence and cholera-infected flies and fleas in opposition to the Chinese (Novick and Marr 2003). Throughout the Cold War, the USSR evolved numerous methods for using ticks to spread diseases like foot and mouth disease; however, they did not use them against any nation (Ban 2000). Similar to this, the USA established a laboratory during the Cold War that may generate millions of yellow fever-carrying mosquitoes to assault the Soviet Union. Additionally, they carried out experiments on the mosquito’s ability to endure a fall from an airplane (William 1981).

### After the First World War

Actually, it is commonly indicated that before sending livestock into enemy states, the Germans vaccinated them against *Pseudomonas mallei* and *Bacillus anthracis*, which can cause serious illnesses like glanders disease and anthrax (Poupard and Miller 1992). It was expected that biological weapons would be used more extensively in WWII, given the widespread use of non-conventional chemical weapons in the first war. Several countries carried out research projects to create bioweapons during this war; the Japanese program, which produced a bioweapon, was thought to be the most ambitious (1892–1959). Lieutenant General (Lt. Gen.) Ishii began conducting research in this area in 1928 after traveling to several European and American nations to pick up practical skills and knowledge regarding the potential applications of biological weapons. Upon returning to his native country, he received a sizable grant to establish a large bioweapons research facility in Beiyinhe, Manchuria, known as Unit 731. Approximately 3000 scientists, primarily microbiologists, worked at the research center and carried out the experiments on prisoners of war, primarily soldiers from China, Russia, and Korea. Numerous bioweapons, such as *Yersinia pestis*, *Vibrio cholerae*, *Neisseria meningitidis*, and *Bacillus anthracis*, were tested on the prisoners (Ban 2000). Many thousand prisoners died as a result of the experiments carried out on them during this research. However, because of the actions taken by Lt. Gen. Ishii, the death rate in the vicinity of Unit 731 continued to be exceptionally high for several years, with as many as 200 000 fatalities (Harris 1995). In 1942, 1700 Japanese soldiers lost their lives because of the inadequate number of other countries experimenting on hypothetical biological agents. The British Army’s 1942 experiment off the coast of Scotland, on the Island of Gruinard of the Scottish coast, was the site of anthrax bomb tests (Manchee et al. 1981).

### After the Second World War

The USA lagged well behind other countries in bioweapon (BW) research up until WWII. The heyday of both product development and testing BW in the USA began right away following the Second World War when it was given the findings of the Investigations carried out by Japanese Unit 731 (Christopher et al. 1997). As reported, in September 1950, the US Navy tested civilians to determine how susceptible a sizable coastal town in the USA was to a bioattack in the San Francisco Bay Area. Mostly, a mist of low pathogenic bacterium *Serratia marcescens* accountable for the infections of the skin and respiratory tract was dispersed by boat, starting the infection and spreading, as verified by additional investigations, which comprised nearly the whole populace (one million individuals). Although the bacteria were essentially innocuous, multiple acquired respiratory conditions, and few even passed away (Christopher et al. 1997). According to different reports, between 1956 and 1958, large numbers of mosquitoes in Georgia and Florida were likely carrying Yellow Fever, which was disseminated to confirm airborne sensitivity assault. Top secret status applies to the documents according to several accounts; some people died from the insects that bite people and carrying the yellow fever. A large-scale trial conducted in the USA was associated with the deliberate release of the harmless bacterium *Bacillus subtilis* in the New York City subway system during the summer of 1966. Although the experiment resulted in some infections, there were no serious health consequences among the more than one million individuals potentially exposed. The trial demonstrated that a pathogen released at a single station could disperse throughout the entire subway system via airflow in the tunnels (Pal et al. 2017).

## Agents of biological weapons

### Microorganisms

#### Bacillus anthrax

**Fig. 1:**
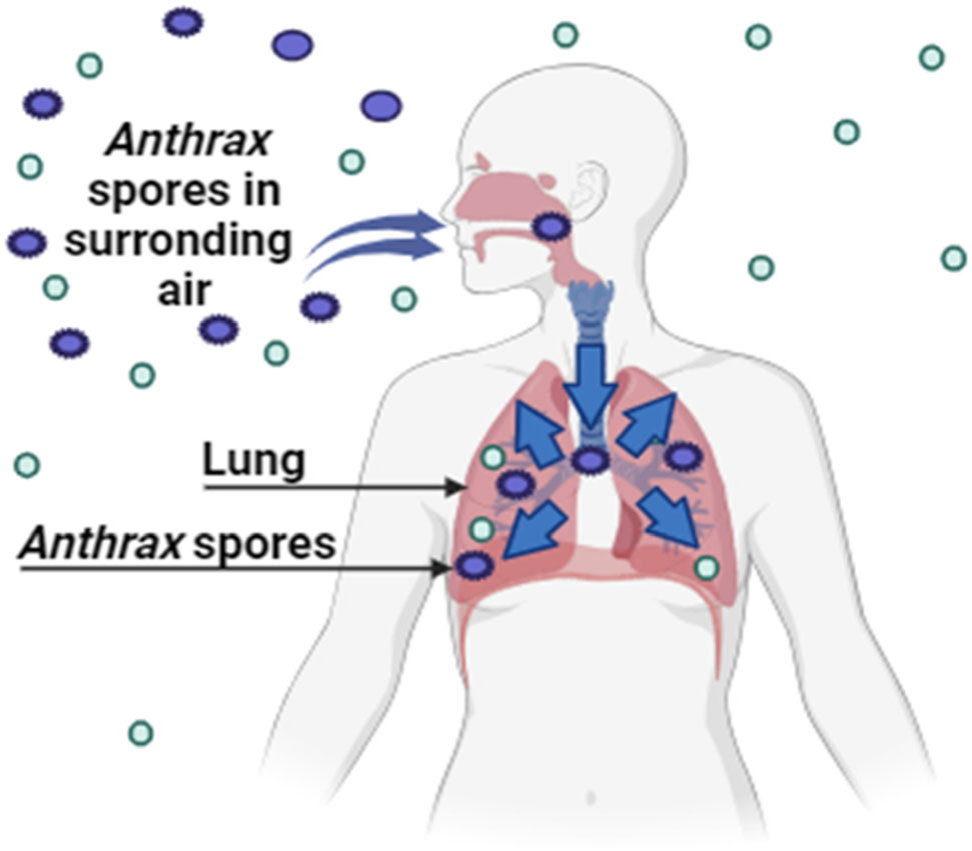
Pathway of anthrax spores after human inhalation. (Created with BioRender)

*Bacillus anthrax* is a gram-positive, facultative anaerobe, encapsulated non-motile bacteria that produces spores. The most sought-after biological warfare agent is spores due to their extreme resilience to environmental stress. In both water and soil, sporulated cells can endure for many years. Gruinard Island was permanently contaminated because of the US and Allies’ 1943 live-fire testing of anthrax bombs there. Only after employing formaldehyde as a disinfectant for more than 40 years was decontamination possible. There are three forms of anthrax, a disease mostly affecting animals and brought on by *Bacillus anthrax*: (i) cutaneous anthrax, (ii) gastrointestinal anthrax, (iii) respiratory anthrax (Harris 1995) (Fig. 1).

If left untreated, respiratory anthrax has a death rate of over 80%, making it the most severe variety of the disease. The World Health Organization (WHO) estimated that 500 000 persons would be impacted and that 100 000 of them would die if an airplane dropped 50 kg of anthrax spores (Morgan 2000). The permissible detection limit for these approaches has been as low as 1–1000 spores found. Although it can be challenging to identify spores from environmental samples, luminescence microscopy in conjunction with before concentration is a quick, simple, and accurate technique. A few cutting-edge portable tools, such as the biohazard detection system (Meehan et al. 2004) and rapid measurement platform (RAMP) (Du Plessis 2017), have also been created to identify spores of *Bacillus anthrax* in ambient material samples. Many nations utilize protein-based and live attenuated vaccinations for humans, but these immunizations also have adverse effects and only offer temporary protection. Tetracycline and other popular antibiotics are often effective when an infection is still in its early stages, but once symptoms appear, treatment is no longer helpful (Harris 1995). The most popular form of bioterrorism is inhalation, which necessitates aerosolization and is difficult due to the requirement for specific technological procedures to grind the spores into a fine powder. Delivery is simple once the proper form is taken, and commercial aerosol products can help. In the 1950s and 1960s, the USA began using anthrax spores as a weapon before discontinuing its previous offensive program. Other countries have weaponized this agent, or they may have done so. Iraq admitted researching the offensive use of *Bacillus anthrax* prior to the Persian Gulf War to a United Nations inspection team in August 1991. In 1995, Iraq acknowledged turning anthrax into a weapon. Anthrax can be produced in a wet or dried form, stabilized for weaponization by an adversary, and delivered as an aerosol cloud either from a line source, like an aircraft flying upwind of friendly positions or as a point source from a spray device. A recent biological weapons program defector from the former Soviet Union disclosed this information. According to Centers for Disease Control and Prevention (CDC), a large ground area could potentially be covered theoretically by multiple spray bombs fired from a missile battle head at a fixed height above the ground (Centers for Disease Control and Prevention 1999). However, in 2001, the intentional distribution of *Bacillus anthrax* spores via the postal system resulted in an unprecedented biological attack that began in the USA in mid-September. A number of prominent Americans, including Senator Tom Daschle, Tom Brokaw, and the New York post offices, received letters containing anthrax spores (Centers for Disease Control and Prevention 1999). This bioterrorist operation has not yet been fully quantified, but it has already cost a great deal. Hundreds of people were affected. In a twentieth-century series of cases, the fatality rate from occupationally acquired inhalational anthrax was 89%; however, the majority of these cases occurred prior to the establishment of critical care units and the development of antibiotics (Roberts 1993).

#### Yersinia pestis

**Fig. 2:**
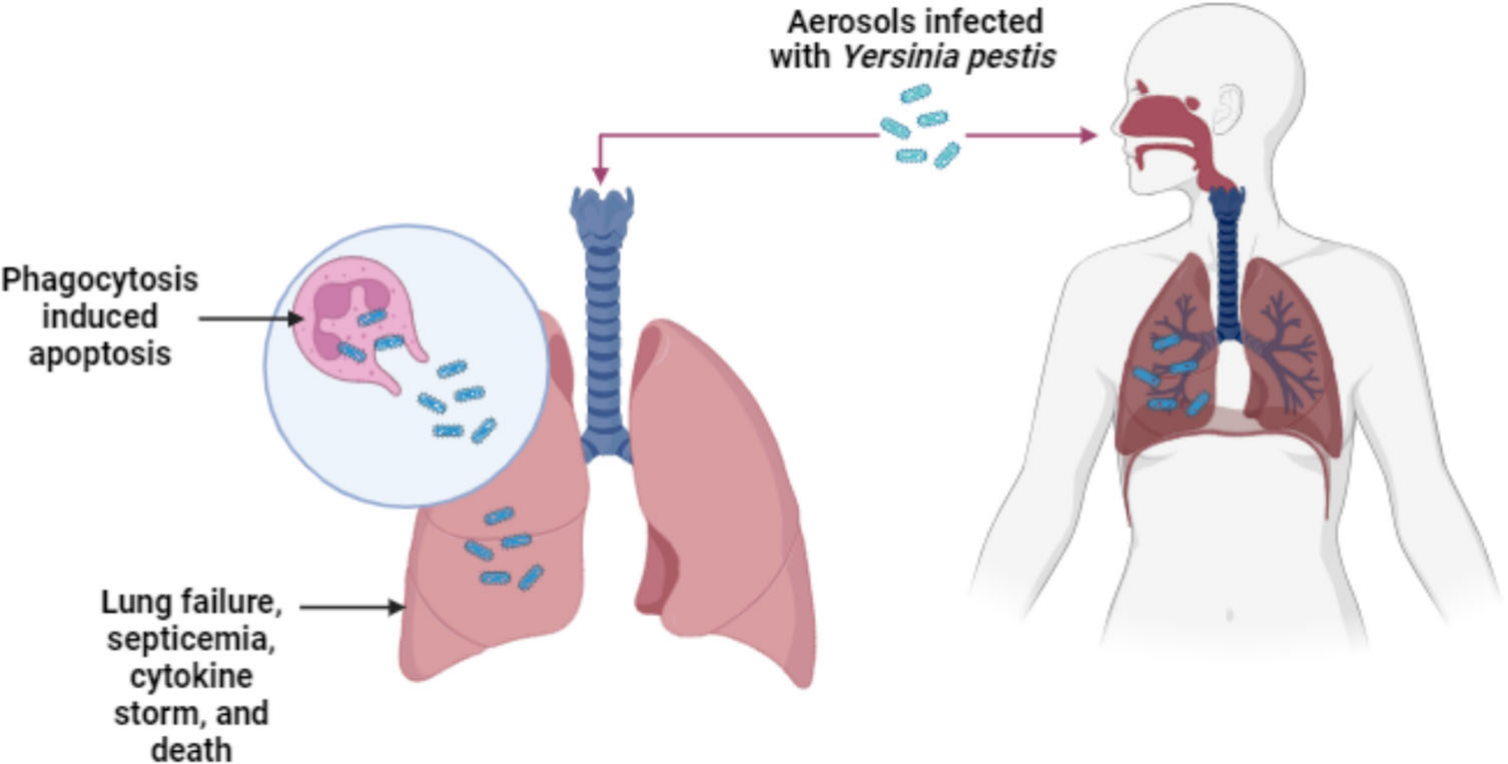
Schematic representation of *Yersinia pestis* transmission and migration in the human inhalation. (Created with BioRender)

A facultative anaerobe that is gram-negative, non-motile, and rod-shaped, displaying bipolar staining, commonly referred to as “safety pin” staining when using Giemsa stain. This bacterium is the causative agent of the plague, a zoonotic disease that affects rats and other animals, and is usually transmitted to humans through flea bites. The bubonic form of the plague, resulting from this transmission, is characterized by swollen lymph nodes in the armpits and groin, known as buboes. Pneumonic plague can develop from inhaling the bacteria or through hematogenous spread from the lungs to the buboes during a bioterrorist attack. Pneumonic plague is highly contagious, spreading via aerosol droplets from person to person (Fig. 2). Early treatment with gentamycin, streptomycin, or tetracycline can be effective, but untreated cases have a death rate exceeding 50% (Bhushan and Katyal 2002). Aside from smallpox, it remains a significant biological threat beyond initial infection. Containing a plague epidemic is challenging in today’s era of air travel. This pathogen poses severe risks to human health, and despite advancements in antibiotic treatments, its potential as a bioweapon persists (Perry and Fetherston 1997). Historically referred to as the “Black Death,” the plague is still primarily a bubonic disease spread by fleas, but recent developments suggest the emergence of a pneumonic variant that is significantly more contagious. Past reports indicate that direct exposure to aerosolized plague bacteria is the most effective method for infecting humans and causing fatalities. Both the US and the former USSR explored the feasibility of aerosolizing the plague as part of their biological weapons programs (Frist 2002). Although the Soviet Union had missile warheads containing plague bacillus prepared for deployment before 1985, significant challenges remained in producing and dispersing large quantities of the bacteria using modern weaponry (Ban 2000). Nevertheless, the plague is still regarded as a high-risk agent for bioweapon development (Arnon et al. 2000).

#### Burkholderia mallei

**Fig. 3:**
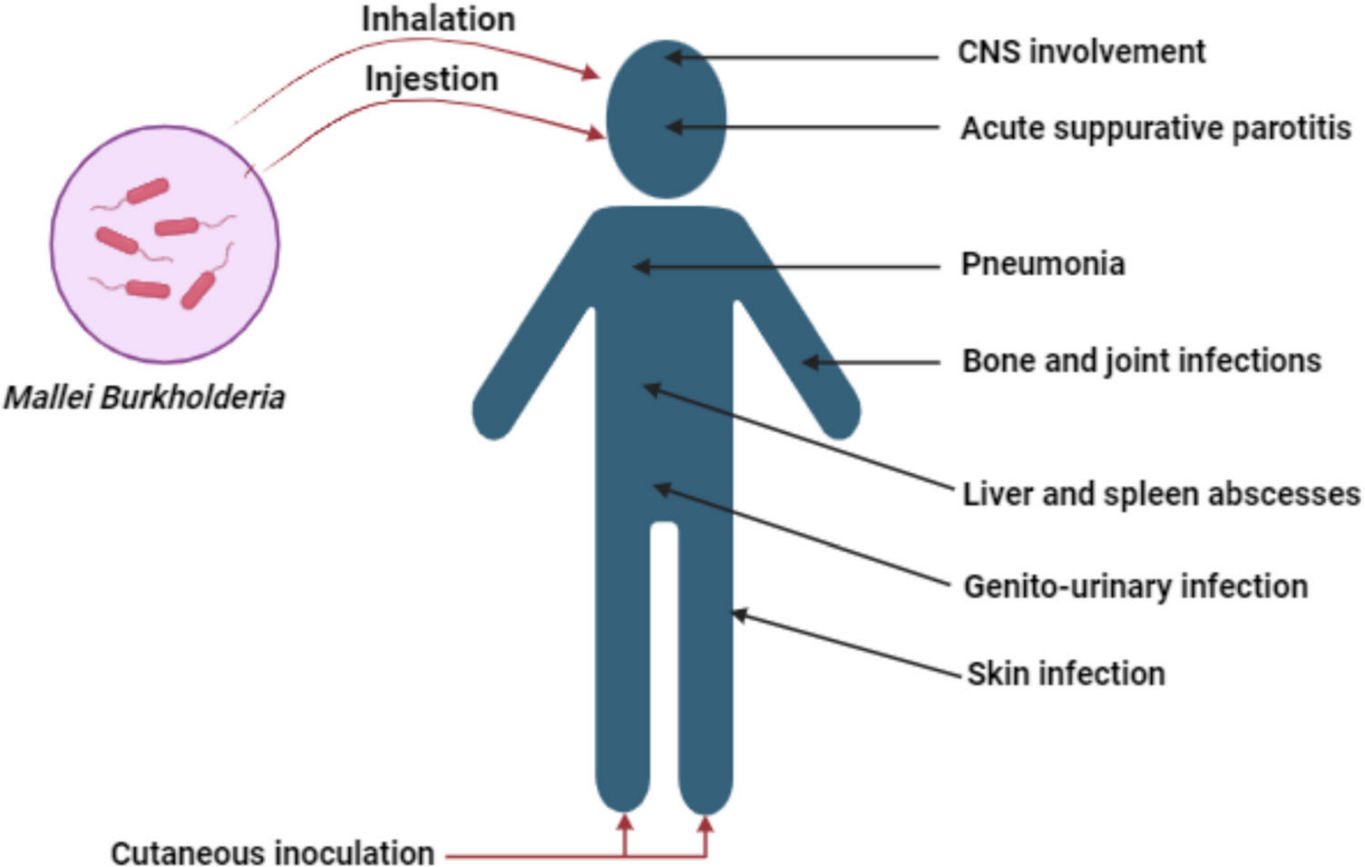
Insights into the pathogenicity of *Mallei Burkholderia*. (Created with BioRender)

The bacterium *Burkholderia mallei*, rod-shapped non-grammatic bacteria, is the cause of glanders, a feverish disease that primarily affects horses. Their unintentional hosts are humans, and cases of the illness have been documented among those who have handled sick animals. *Burkholderia mallei*is thought to have the potential to be a biological warfare agent because of its low infectious dosage, lack of a viable vaccination, and treatment (Fig. 3). Four kinds of glanders can manifest: an acute, localized form, a deadly, septicemic form two types of pneumonia: acute and chronic (Christopher et al. 1997). Any of these four kinds can be caused by aerosol infection. The symptoms of glanders, which comprise fever, sweating, muscle soreness, headaches, edema, and chest pain in the lymph nodes, purulent outbursts, and other symptoms, often appear 10–14 days after exposure. If left untreated, the septicemic variant has a 100% mortality rate, although transmission from one person to another is uncommon (Detrick and Frederick 2001).

#### Francicella tularensis

The tularensis infection is caused by gram-negative coccobacilli, primarily *Francisella tularensis*. This is a zoonotic illness, meaning humans contract it from animal or insect bites. Even though the organisms are heat sensitive, they can survive for extended periods in the environment and animal products. In humans, inhalation or ingestion of the bacteria is rare in natural settings; however, aerosol exposure (such as in a biological warfare attack) can lead to pneumonic tularemia. Because the illness can be triggered by as little as 10–50 organisms, *Francicella tularensis* is a desirable biological warfare agent. It can take two to ten days for it to incubate. Inhaled pneumonic tularemia can cause fever, headaches, exhaustion, weight loss, active coughing, and pneumonia. Antibiotics can treat tularemia, but if left untreated, the death rate could range from 35 to 60 percent (Harris 1995). Early in the nineteenth century, tularemia was identified in Russia and in Japan in 1826. Growing and developing these bacteria is challenging, but it is possible to separate tularemia from affected organisms and use it as a biological weapon. Furthermore, it can easily spread through aerosol emissions and has been used as a weapon by the US and the USSR in the past. In a number of situations, tularemia may be employed as biological weaponry with differing degrees of fatality. A high number of people exposed to aerosol emission is the deadliest scenario. More problems would occur if an antibiotic-resistant strain, which is believed to have originated in the former Soviet Union, was used (Alibek and Handelman 1999). A widespread 50 kg aerosol release over a large metropolitan area could render 250 000 people incapacitated, according to research. Pneumonic tularemia-like pulmonary symptoms may follow a nonspecific febrile illness that most affected individuals may experience 3–5 days after exposure, depending on the exposure inoculum. Nonetheless, it may take longer to identify tularemia as the underlying cause due to the challenges in diagnosing the illness and its vague clinical manifestation. It may be challenging to differentiate cases at first from other respiratory infections or a spontaneous influenza outbreak (Dennis et al. 2001). It is possible to mistake tularemia for another biological weapon. Large pleural effusions and mediastinal widening, which are indicative of inhalational anthrax, may be observed on chest radiographs as a pattern of pulmonary manifestations. The clinical course of tularemia is slower, and the case fatality rate is higher with plague (Inglesby et al. 2002). These epidemiological hints can be used to distinguish tularemia from anthrax or the plague. Differentiating pulmonary tularemia from *Q fever*, another potential biological weapon, may prove to be difficult (Inglesby et al. 2002).

#### Clostridium botulinum

The four identified species of *Clostridium botulinum*, which are identified by their unique genomes and shared botulinum toxin, are the etiological agents of botulism. It is an obligate anaerobe that forms spores and can be isolated from the soil, which is its natural habitat. In addition, the lack of cross-neutralization distinguishes seven different antigenic kinds of botulinum toxin (A–G). The disease-causing toxin is a di-chain polypeptide: One disulfide bond joins a 50-KD light chain and a 100-KD heavy chain. This zinc-containing endo peptidase prevents vesicles carrying acetylcholine from merging with the terminal membrane of the motor neuron, which causes flaccid paralysis of the muscles (Arnon et al. 2000). The most deadly poison known is botulinum toxin, of which there are seven varieties that all function similarly. Paralysis of the diaphragmatic and pharyngeal muscles frequently, which is then followed by respiratory arrest, results in death (Bhalla and Warheit 2004). Due to its high potency and lethality, simplicity in production, transportation, and misuse, as well as the need for prolonged critical care for those who are impacted, botulinum toxin poses a serious threat to bioweapons (US Department of Health and Human Services 2000). A botulism outbreak is both a medical emergency requiring early administration of botulinum toxin and frequent mechanical respiration, and a public health emergency requiring quick action to stop further cases. An observant clinician is the first to identify a botulism outbreak in time and promptly alert public health authorities. The most dangerous material currently known is botulinum toxin (Waters 1996).

### Viruses

#### Smallpox virus

Of all the animal viruses, the most widespread one is smallpox. Virus particles range in form from brick to ovoid. The smallpox virus only naturally infects humans, and the illness spreads from person to person. It causes fever and headaches after an incubation period of roughly 12 days. The virus causes pus-filled vesicles to grow throughout the body when it spreads to the skin. The death rate for those who have had vaccinations is about 3%, whereas it rises to %30 for those who have not. An individual exposed to aerosols may have headaches, backaches, vomiting, fevers, and rigors. The following factors make the smallpox virus a far greater threat (Franz et al. 1997): (i) since smallpox vaccinations were discontinued in 1980, a greater portion of the global population is not protected against the disease, (ii) high aerosol transmissivity, and (iii) how simple it is to culture the virus. As with the smallpox virus, there is no reliable cure, and vaccination is only accessible in the USA. Following the WHO report from 1980, which declared that the world was free of smallpox, people’s immunization programs were discontinued. This left the unvaccinated human population vulnerable to infection by the *Variola virus*, should it arise through bioterrorism or other means. The smallpox virus has no known cure, and the vaccine is only accessible in the USA (Harris 1995).

#### Virus causing venezuelan equine encephalitis (VEE)

The illness is brought on by a mosquito bite and is caused by an arthropod-borne virus. Donkeys, horses, and mules are among the animals that naturally harbor this virus. In addition, this virus spreads easily by aerosols. The illness manifests itself as lethargy, a rising fever, headaches, and light sensitivity through a two to six-day incubation period. As a result, some individuals may experience neurological problems later in the course of the illness**.** It is mostly regarded as an incapacitating agent, with a very low (1%) fatality rate (Detrick and Frederick 2001).

#### The marburg and ebola viruses

Among other viruses, marburg and ebola are the two main ones that cause hemorrhagic viral fever. When human body fluids or organs come into direct contact with one another, these viruses are transferred from one person to another. The low infectious dosage, lack of therapy, and high fatality rate (30–90%) make using these viruses as biological warfare weapons appealing. For viral hemorrhagic fever, the incubation period varies from 4 to 21 days. Fever, headache, sore muscles, vomiting, and diarrhea are among the symptoms (Wannemacher and Wiener 1997).

### Fungi-based agents

The fungus-causing pathogen is the dimorphic spore-forming *Coccidioides immitis*, which causes coccidioidomycosis. Usually, the illness manifests as mild flu-like symptoms. Frequently, the condition begins as a mild, benign, or inconspicuous upper respiratory infection that clears up fast. However, if the infection is controlled, the illness can worsen and develop into a systemic illness that affects the meninges, which cover the lining of the brain, joints, bones, and cutaneous and subcutaneous tissues (Detrick and Frederick 2001). The death rate in instances that go untreated can reach 50%. Iitraconazole, ketoconazole, or amphotericin B can all be used to treat the illness. Even though the majority of fungi rarely kill healthy people, they can be utilized to ruin crops (Cupp et al. 2004).

### Toxins

**Fig. 4:**
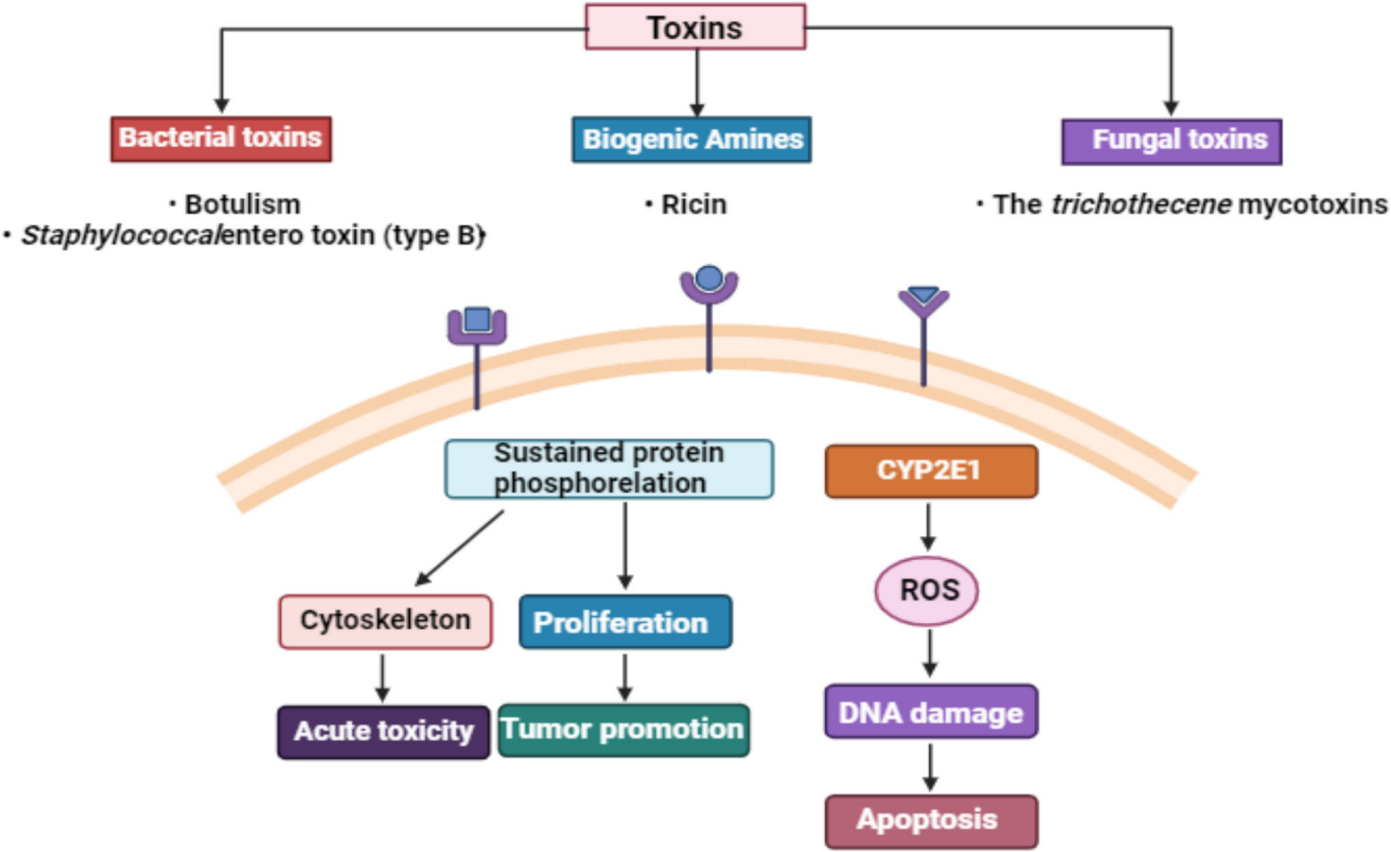
Different types of toxins and their potential in boosting human health. (Created with BioRender)

Aside from ricin (Phytotoxin), three microbial toxins could primarily be employed as agents of biological warfare (Fig. 4).

#### Toxins found in botulinum

These represent the agents that are responsible for the sickness known as botulism, and *Clostridium botulinum* is the bacterium that produces them. When an organism lyzes and dies, these poisons are released. Toxins like botulinum are the worst compounds known to humanity. Even five pictograms (5 × 10–12 kg) of botulinum toxin can be fatal to a laboratory mouse. 39.2 g, roughly, of botulinum toxin would be enough to eradicate the six billion people on the planet. The toxin is 100 million times more toxic than cobra venom and 100 000 times more deadly than sarin, the most feared nerve agent used in chemical warfare (Dhaked et al. 2002); by inhibiting the production of the neurotransmitter acetylcholine, botulinum toxin stops nerve impulses from being sent. Traditionally, botulism in humans manifests as food poisoning without fever, though it can cause breathing difficulties and visual issues (perhaps causing the pupils to be fixed). Respiratory arrest occurs within 24 h of toxin consumption, resulting in death. It is possible to lower the death rate from botulism to 10% with efficient supportive care (Harris 1995). Despite the potency of botulinum toxin, research conducted by Indian scientific institutes appears limited, and bacterial cultures or isolates are reportedly unavailable in India; however, the Defence Research and Development Establishment (DRDE) in Gwalior has successfully isolated *Clostridium botulinum* from the local environment, demonstrating that the capability to study this toxin exists within specialized defense research institutions (Harris 1995).

#### Staphylococcal enterotoxin (type B)

The majority of individuals get food poisoning at some point in their lives, which exposes them to Staphylococal enterotoxin (type B) (SEB), an enterotoxin produced by the bacteria *Staphylococcus aureus*. Aerosolized SEB has the potential to be a biological weapon that could make 80% or more of the targeted personnel very sick in 3–12 h. The toxin has a high degree of resistance to acids and alkalis and can endure boiling temperatures. These qualities raise concerns about the potential for a SEB attack on food and water supplies. It can take up to two weeks for humans poisoned with SEB to recover, and higher doses can even cause septic shock and death (Cupp et al. 2004).

#### The trichothecene mycotoxins

Many different fungi, including *Fusarium*, *Trichoderma*, *Myrothecium, Stachybotrys*, and others, produce trichothecene mycotoxins, also known as T2 toxin. These lead to an illness known as alimentary toxic aleukia (ATA). Vomiting, extreme skin irritation, and internal bleeding are common signs of ATA (Bunner et al. 1985). Among biological warfare agents, T2 toxin is distinct due to its high efficacy for causing skin damage because it is hundreds of times more poisonous than chemical warfare agents like lewisite or mustard (Cupp et al. 2004). Its capacity to harm eyes in microgram concentrations adds more evidence that it is more hazardous than mustard gas (Harris 1995).

#### Ricin toxin

Ricin toxin is produced worldwide by the castor bean plant, *Ricinus communis*. The toxin comes from the plant’s seeds. Ricin is a hazardous substance that can be inhaled, injected, or even consumed. It is largely stable in the environment and is made up of the peptide chains A and B. The toxin’s widespread availability and ease of manufacture serve as its primary justifications in order to employ it as a biological weapon. Within 18–24 h following exposure, symptoms of ricin toxicity, such as fever, coughing, dyspnea, nausea, and joint pain, manifest. When breathed, ricin may cause respiratory failure (Detrick and Frederick 2001). Additionally, if consumed, it results in vascular collapse and damage as well as severe gastrointestinal symptoms. If injected, it causes multiple organ failure. As a prophylactic measure, there is not currently a vaccine against ricin toxicity (Manchee et al. 1981).

## Characteristics of a perfect biological weapon

When compared to the hydrogen bomb, a biological weapon may be more potent. This examination of biological weapons demonstrates an effective agent’s potential impact. A population is intended to be destroyed by biological attacks, either by mass destruction or by causing illness in a huge number of individuals. In order of relative significance, the following desired attributes are listed: highly infectious, extremely hazardous, preferably spreadable among humans, stable during storage and dispersal, difficult for medical personnel to respond to, easily grown, and capable of producing controlled effects (Singha et al. 2020). Few pathogens satisfy the requirements to be categorized as BWs despite the fact that many bacteria, viruses, and poisons cause illnesses in humans, animals, and plants. Eitzen (1997) outlined the qualities that define a biological agent as a possible biological weapon. A BW should ideally be simple to locate or create a biological attack on susceptible targets or the broader public; enormous amounts of biological agents are actually required. It must be remembered that a target must be infected with a disease that can only be caused by a huge number of biological agents or a certain quantity of toxins. Additionally, the perfect BW needs to be extremely fatal or have a great potential to incapacitate the afflicted (Lockwood 2012)**.** It is appropriate to select an agent with an incubation period according to whether you require immediate or delayed effects. A biological weapon’s mode of transmission and, consequently, ease of dissemination, when delivered via the proper mechanism, are additional crucial aspects. Lastly, the agent’s stability needs to be evaluated, particularly if significant amounts need to be kept indefinitely (Kortepeter and Parker 1999).

## Distribution and delivery methods for biological weapons

Delivery methods for biological agents include both moist and dry forms. Dry powders with tiny particle compositions typically have superior dispersion properties and benefit from storage advantages. While spray-drying and freeze-drying technologies have been around for a while, the production of dried agents requires a higher level of technological complexity. Aerosolized agents are the most often used delivery techniques. The agent can be dispersed using a spray device that is affixed to a moving vehicle. There will be a release of line while the sprayer is operating. This is sprayed perpendicular to the direction of the wind and is referred to as a line source. Inland from the designated target region up to a certain distance, anyone downwind of such a line source would theoretically be at risk (Arnon et al. 2000). The range of an infectious or toxic agent depends on a number of variables, such as the agent’s characteristics, wind direction and speed, atmospheric stability, and the existence of inversion conditions. Biological agents can spread through food and water contamination, animal infection that infects humans, and airborne spraying. Numerous human pathogens have the potential to be armed. Only a small number, nevertheless, have been recognized by public health authorities as potentially causing widespread deaths and upsetting social. Biological weapons have the potential to cause more widespread casualties and disruptions to civil unrest for a number of reasons. Utilizing easily accessible instruments such as industrial sprayers or other devices that generate aerosols, a perfect biological warfare agent could be easily spread outdoors. Biological agents are released into the atmosphere as aerosols, creating a fine mix (Zilinskas 1990). The most efficient way to deliver biological warfare agents is via aerosol. The goal of aerosol delivery systems is to create invisible clouds made up of droplets or particles that are between 0.5 and 10 µm in size. Because the respirable particles in that size range can settle deep in the lungs, the primary hazard from the aerosol release of those particles is inhalation. It is possible to use biological warfare agents to contaminate water or food supplies. Most agents need to be applied to food that will be served raw or added after the food is prepared and presented for serving in order to be effective because heat destroys the majority of pathogens and toxins. Numerous pathogens and toxins may be rendered inactive by using conventional water purification techniques like chlorination and filtration. Chlorination, on the other hand, will not kill off a lot of spores, and commercial filtration will not do much to stop viruses, spores, cysts, or many bacteria. When it comes to eliminating toxins, filtration is nearly ineffective unless activated charcoal is employed. Covert injections have been used to deliver biological warfare agents. Certain substances like ricin, for instance, can be fatally injected. Depending on the type of delivery system being used, the time of day, the weather, and the topography of the area, there are many different potential BW attack methods in any operational environment (Pinson et al. 2013).

## Effects of exposure to biological weapons

Many methods exist for the spread of biological agents. The following are the modes of transmission: inhaled substances administered by blood or bodily fluids; droplets in the airway; substances that afflicted people release into the air; after that, nearby individuals may breathe them in. Contact is the process by which agents on an infected organism’s surface can spread to another organism. The oral–fecal pathway involves intercourse or coming into contact with objects, foods, or other things tainted with infected patients’ excrement (Whittier 2017).

### On the breathing system

The most dangerous is inhalation, which resembles flu symptoms but causes respiratory failure and lung fluid buildup. The vast surface area of the lungs and the ability to exchange gases make the body particularly vulnerable to this type of exposure due to the mucous membranes’ vulnerability to infection and the existence of phagocyte cells, which, if they are unable to eradicate a pathogenic microbe, they might instead transfer it to the lymphatic system, where it could grow and damage the lungs from the majority of biological agents. Aerosol particles, as opposed to vapors, build up in the respiratory system over time at a specific size (Steffen et al. 1997).

### On the skin and mucous membranes

The least dangerous type of skin cutaneous enters through cuts or abrasions and leaves a lesion with a black scab. This could be drastically altered by the presence of wounds, sores, or skin rashes, which could even provide a pathway for biological agents to enter the body. Skin is generally more vulnerable to penetration; it is thinner, more vascular, and moist. Elevated relative humidity encourages skin absorption. Aerosols and liquid spills can pose a multiple delivery risk for skin penetration. When using improvised devices, a greater percentage of the agent will be spilled, and a smaller portion will typically be vaporized at a higher order of magnitude. The main area where spills will occur is near the aerosolized point (Polhuijs et al. 1997).

### On the system of digestion

Antibiotics can be used to treat gastrointestinal issues such as eating contaminated meat or water, nausea, bleeding and discomfort in the abdomen, fever, and throwing up. Biological weapons can enter the body by ingestion of respiratory mucus that has accumulated after larger aerosol particles accumulate in the upper respiratory tract, nose, and throat or by contaminated food, water, or surfaces that are touched by the hands and mouth. If the contaminated sources are identified, this exposure route can be controlled the most easily out of all of them (Polhuijs et al. 1997).

### Effects of bioterrorism on humans and animals

A small-scale biological attack on an urban center with a weapon-grade agent could cause a great deal of morbidity and mortality, far exceeding the capabilities of the local medical system. For example, it has been calculated that up to three million people could die from an aerosolized release of as few as 100 kg of anthrax spores upwind of a metro area the size of Washington, DC. Additionally, it causes a great deal of animal morbidity and mortality as well as financial loss (Wannemacher and Wiener 1997). A lot of the drawbacks of using biological weapons have to do with how hard it is to carry out an attack. The use of weapons is related to the challenges of carrying out an attack. For instance, it can be challenging to safeguard employees throughout the production, shipping, and delivery processes, and vaccinations may be ineffective or unnecessary. The risk that biological agents may also have an adverse effect on the health of the attacking forces; the way that effective dispersion depends on the dominant winds and other weather factors; the effects of temperature, sunlight, and desiccation on the survival of some infectious organisms. Additionally, the potential for an agent to produce secondary aerosols as the aggressor passes through an area that has already been attacked, the ability of some agents, like the spore-forming *Anthrax bacteria*, to persist in the environment and render a region uninhabitable for extended periods of morbidity linked to a biological attack (Polhuijs et al. 1997)**.**

## Entomological warfare

The practice of using insects as disease vectors is known as “entomological warfare.” Although biological warfare is the primary application for these insects, they can also be employed as living dirty bombs to disperse chemical or radioactive materials. Actually, by being aware of their habits, one can select the right insect to deliver a chemical, biological, or radioactive agent to particular targets in an effective manner and employ insects to harm adversaries (Peterson 1990). In order to achieve this, pathogen-infected insects are first released into the intended target area. These insects serve as vectors, infecting specific animals or residents of the intended location. Insects of the second kind are utilized directly to harm crops. In the third category, uninfected insects such as bees are deployed to attack enemies directly (Lockwood 2008).

### Entomological warfare in past

The entomological war has been used to disseminate illness in the past; Europeans used bees to defend their city against an Austrian force in 1289 (Ambrose 1974). Septimus Severus, a Roman emperor, tried to rule Mesopotamia at the close of the 2000s. The city of Hatra was one of the primary targets for control of the major trade routes in modern-day Iraq. Aware of the impending Roman invasion, the Hatrians prepared earthenware containers with stinging arthropods inside of them (Roland 2005). In 1799, the Ottoman Empire declared war against France, which prompted Napoleon Bonaparte to send 13 000 soldiers into Syria. Upon reaching Jaffa, the French soldiers fell ill with the bubonic plague (Peterson 1995). Native Americans in the 1800s employed ants to cause excruciating, long-lasting death before being staked over anthills; victims had their mouths held open with skewers or had honey applied to their lips and eyes (Wellman 1987). Numerous pathogens and vectors were created at Camp Detrick, such as ticks carrying tularemia, Colorado fever, and relapsing fever flies carrying cholera, anthrax, and dysentery, as well as mosquitoes carrying dengue, malaria, and yellow fever. The 1950s saw the height of American Entomological Warfare efforts as covert field tests and laboratory research produced results (Lockwood 2008). Methodically, "scattering large quantities of bacteria carried by insects by aircraft in order to disseminate infectious diseases" is the official charge made by North Korea against the US military in February 1959 (Croddy et al. 2002). In the decades that followed, Fidel Castro charged that the USA was engaging in entomological warfare, but there was no solid proof until 1981. One hundred fifty-eight people died, and 344 203 cases of dengue fever, a mosquito-borne illness, were reported during a five-month outbreak. A gray mist was released over Cuba by a US State Department plane in 1996. The pilot saw a Cuban plane, according to the Americans, and signaled with smoke to make sure he could see it and the smoke was actually a cloud of *Thrips palmi*, which led to an infestation of the pest, according to the Cuban government (Lockwood 2012).

### The risk of insects in warfare

**Fig. 5:**
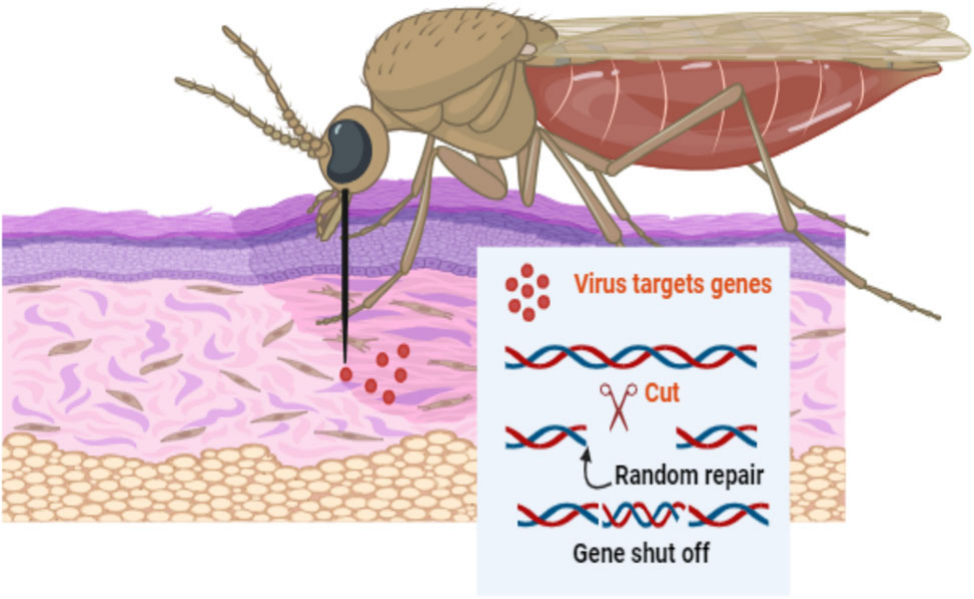
Schematic diagram show genetically modified viruses transmitted by insects have the capacity to modify chromosomes in infected cells. (Created with BioRender)

Employing insects as weapons carries a high risk. Because so few people have the necessary training to recognize risks, comprehend them, and take appropriate action to reduce them, the issue is barely felt and challenging to handle. The areas that present the greatest challenges are ambulance transportation, rescue, and hospitals that require treatment and diagnosis. However, the target industries that are most likely to be attacked are agriculture and public health in general. The most successful strategic goal is terror, which can be used to deter people from going into an area that is infested with infected insects, cause mass psychosis in specific regions, or cause diseases. We have witnessed how Ebola terrorized anyone suffering from a flu-like illness throughout Europe, even as benign as the flu, as it was hard to distinguish between it and the Ebola virus due to its similar appearance. Ship and aircraft transportation of cargo is undoubtedly a powerful means by which vector-borne diseases spread to new parts of the globe. It is simple to use the freight transport system to transfer insects into new areas or to target states because there are few controls and inadequate anti-weeds measures in place. One or more insects in a suitcase can easily go unnoticed due to their difficulty in detection, and it is unlikely that their use as pathogen incubators will be suspected. Italians are currently experiencing psychosis related to the Zika virus, which is spread by mosquitoes; because of the Zika virus, the WHO has declared an international public health emergency (Fig. 5). So we are reviewing a report from the US army that describes the military’s interest in using mosquitoes as weapons (Ban 2000).

### Entomological terrorism

Three main categories can be used to classify entomological terrorism: using insects as direct attack weapons, agroterrorism agents, and disease vectors (Lockwood 2008). The emir of Bukhara, or modern-day Uzbekistan, Nasrullah Bahadur Khan, tortured insects in the middle of the 1800s. The well-known case of two British prisoners (Lockwood 2012) exposed a torture chamber that was equipped with sheep ticks (likely *Dermacentor marginatus*) and assassin bugs (*Reduviidae*) and measured 7 m deep. The pit was covered with an iron grill. After two months in the pit, the jailer for the emir claimed that “masses of flesh had been gnawed off the prisoners’ bones” (Clarke 1968). Since the USDA initially expressed concerns about the country's susceptibility to pests such as the Mediterranean fruit fly (*Ceratitis capitata*) and the khapra beetle (*Trogoderma granarium*) in the early 1960s, other pests have also raised alarms. These include several species of potato weevils, the sun pest (*Eurygaster integriceps*), the durra stem borer (*Sesamia cretica*), and the Asiatic rice borer (*Chilo suppressalis*). These developments have heightened concerns about the potential for agricultural terrorism (Clarke 1968). Numerous diseases carried by insects continue to pose a severe threat to the US citrus industry (Lockwood 2008). An Air War College report included a scenario where saboteurs released grape phylloxera *(Phylloxera vitifoliae*) to cause damage valued at one billion dollars (Kadlec 1998). The report also explained how Pakistan’s cotton crop could be attacked by insects, which would upset the political and economic balance in a strategically important area. Additionally, it was proposed that the biological and ecological characteristics of the introduction of *Bemisia tabaci*, or sweet potato whitefish, in 1991, which negatively impacted California agriculture to the tune of $300 million, were indicative of a covert attack (Kadlec 1998). It is possible to use genetically modified vectors or insect-borne pathogens for entomological terrorism. For Detrick, research focused on developing insect strains that were more resistant to insecticides (Endicott and Hagerman 1998) and had higher biting activity (Hay 1999). A USDA medical entomologist recently asserted that HIV could be transmitted by mosquitoes through genetic engineering (Lockwood 2008). Additionally, a very creative scenario explained how a plant virus spread by white people could be genetically altered to produce botulinum toxin, which would kill a large area of corn (MacKenzie 1999). The most well-recorded instance of entomological terrorism occurred in 1989. A group of domestic eco-terrorists who called themselves “The Breeders” and opposed insecticides revealed that they had been distributing and growing med flies throughout Los Angeles (Lockwood 2008).

### Breeding of infected vectors

How do they grow in a natural reservoir? Pigs, for instance, are easy to breed in a secluded and closed space. To keep bugs out, all you need is a closed space with sealed windows and doors. If the pathogen is not fatal, the living animal reservoir can withstand infection for an extended period and can even reach maturity. It is feasible to introduce insect vectors into this small space, and once the blood meal is made, those vectors will begin to reproduce and support the life cycle of the pathogen. It is sufficient to incorporate everything required for the vector’s life cycle, such as a water cistern for mosquito larvae development, into the restricted environment to ensure the vector’s life cycle. However, the animal reservoir serves as the nutritional source. The culture system is very affordable and low maintenance. The only actual costs are food for the animal reservoir, fruit for a fruit bat, or additional supplies for a pig. It is a very simple project that can be completed for a few hundred Euros. The identification of the contaminated reservoir is the only relevant expense. A veterinarian who monitors livestock from third parties can easily identify an infected animal and locate infected biological material directly from the animal to contaminate a healthy one, thus reducing costs. Verifying the infection and initiating the production of infected vectors will suffice at this stage. A system for gathering simple carriers needs to be added to the cultural system.

Suction hoods can be used to capture flying insects like mosquitoes. At the same time, direct withdrawal is required for walking insects (arthropods). Glass jars that enable the carrier to be released with a single launch make the transport process simple. The hypothetical terrorist can overcome his fear of mosquitoes. At the same time, direct withdrawal is required for walking insects (arthropods) (Lindquist 1987). Glass jars that enable the carrier to be released with a single launch make the transport process simple. The hypothetical terrorist can overcome his fears when a jar is launched, providing him with the psychological stability he needs to flee danger safely. One visitor, such as a pig, fruit bat, or rodent bat, can be moved around the state without raising any red flags. The sedated animal is transported by car or truck with complete conspicuity, and it is nearly impossible to identify the pathogen infecting it or speculate about its potential use as a reservoir (Endicott and Hagerman 1998). Especially if it is lawful to transport animals, it is impossible to identify any biological material that has been transported infected and infects a healthy animal. The biological sample can be preserved in frozen material for several hours, allowing it to be transported across the nation to any “lunch basket” vectors that qualify, such as cockroaches, fleas, ticks, and mosquitoes (Zilinskas 1990)**.**

### Insects radioactivity

Isotopes are used as markers for plants or food sources for bugs. Since 1964, by utilizing radiation and isotopes, the Joint Division of the Food and Agriculture Organization (FAO) and the International Atomic Energy Agency (IAEA) have been managing insects (Lockwood 2008). It is feasible to employ insects as “living Radiological Dispersal Devices (RDDs)” in addition to using isotopes as a marker. It is as easy as eating a meal that has been enhanced with radioisotopes. An illustration would be injecting an animal with a high dose of radioisotopes into its blood before it is stung by hematophagous insects. It permits a large number of insects to absorb radioisotopes readily; the quantity in the blood meal will be a portion of the inoculum diluted in five to seven liters of blood plus the tissue-absorbed loss (Capinera 2020). The insect you choose will mostly depend on the kind of panic you wish to create in the population. Suppose you want to instill fear in the population. In that case, you can choose an erratic and common species like mosquitoes or a more infesting species like cockroaches, which are better suited for enclosed spaces, or ticks, which are better suited for meadows and gardens. Furthermore, at the time of release, there is a chance that any insect could become contaminated. Depending on the approach, it can vary in difficulty, but in certain situations, it can be obtained quite easily. Should the insects perish nearby or excrete radioactive waste (William 1981).

### Various techniques for utilizing insects as biological weapons

Three different ways exist for using EW (Lockwood 2008). In order to achieve this, insects are first exposed to pathogens and then released into the intended target area. These insects spread disease to specific animals and people in their intended area by acting as vectors. Insects of the second kind are used to destroy crops directly. In this instance, pathogens or their vectors may not harm insects. In the third type, enemy insects such as bees are directly attacked by uninfected insects (Lockwood 2008).

### Insect orders in biological weapons

#### Coleoptera

**Fig. 6:**
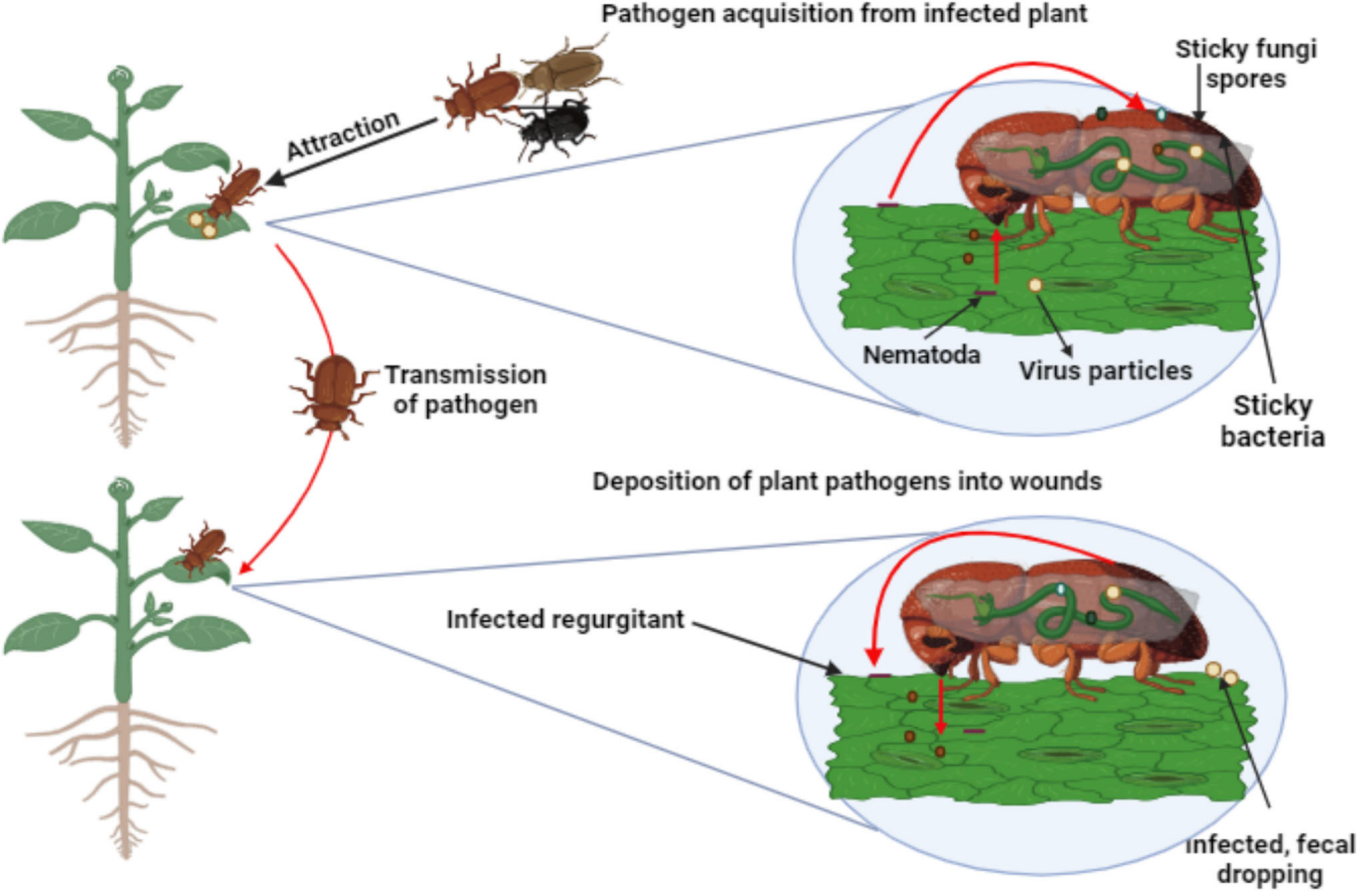
Beetles as plant pathogen vectors. (Created with BioRender)

Coleopteran female can deposit up to 800 eggs, making it a major pest of potatoes. Its elytra have five strips, and its body is orange and yellow (Capinera 2020). As time has gone on, the Colorado potato beetle has developed resistance to other significant pesticide classes, including dichlorodiphenyltrichloroethane (Arnett et al. 2002; Alyokhin et al. 2008; Heather and Hallman 2008); Germans also produced a lot of Colorado potato beetles to destroy enemy food supplies (Lockwood 2008). 54,000 *Colorado beetles* were released in south Frankfurt to see what would happen (Volkov and Baysha 2024). The Colorado beetle is a biological weapon that can be used against enemy crops, according to findings by French entomologists (Lockwood 2008). It was also alleged that the CIA used it against Soviet crops during the Cold War (Farhang-Azad et al. 1985). In order to study Colorado beetles for use as a biological weapon, the USA sent 15 000 of them to Britain in 1942 (Heather and Hallman 2008). The native plants of the Solanaceae family are the food source for the Colorado potato beetle (*Leptinotars decemlineata*) in its range in Mexico. Throughout the second half of the 1800s, the beetle species traveled 100 km annually throughout the United States. The beetle may have been unintentionally brought to Europe during World War I through a food shipment to Bordeaux, France, the primary port for resupplying US forces stationed there. At the Poudrerie National laboratories in Le Bouchet, the French army surreptitiously developed techniques to mass rear the Colorado potato beetle for use as a weapon during the early stages of WWII; they were having previously observed the impact of this pest. This idea served as the foundation for a biological warfare program that the German occupation forces developed after they learned about the laboratories. German scientists looked into the similar propagation of at least fifteen insect species during WWII because of their potential to seriously harm crops and have an economic impact on adversarial nations such as Great Britain (Kotwal and Yadav 2021) (Fig. 6).

#### Siphonaptera

**Fig. 7:**
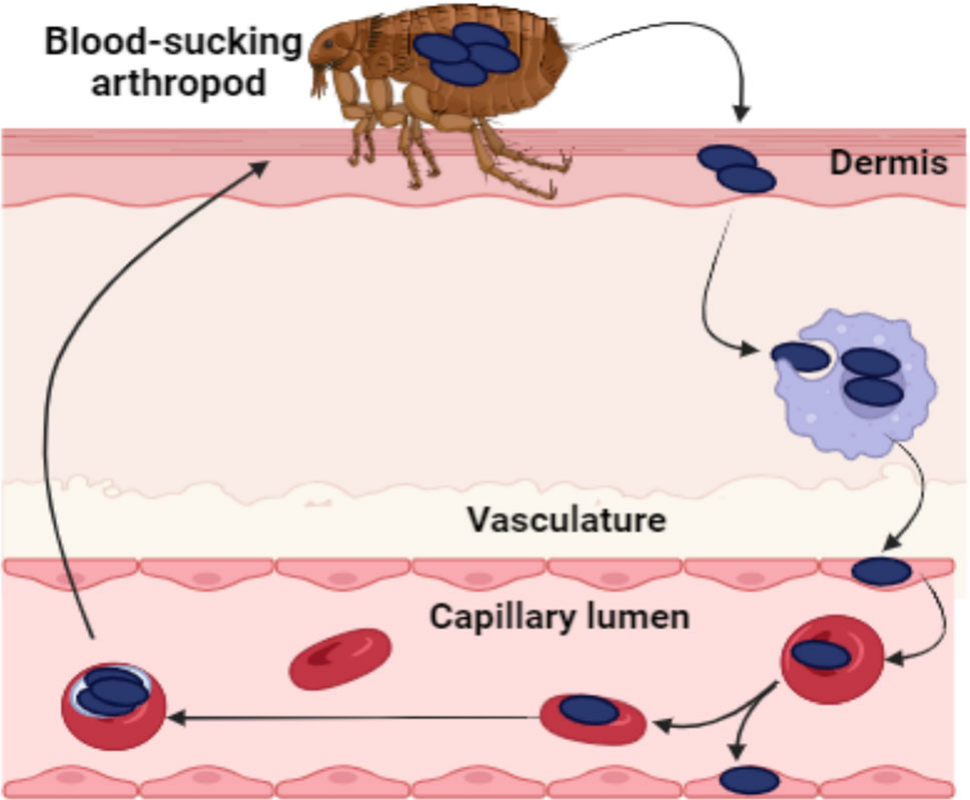
Schematic diagram of transmission of the bubonic plague and murine typhus by the eggs of fleas from one generation to the next. (Created with BioRender)

**Fig. 8:**
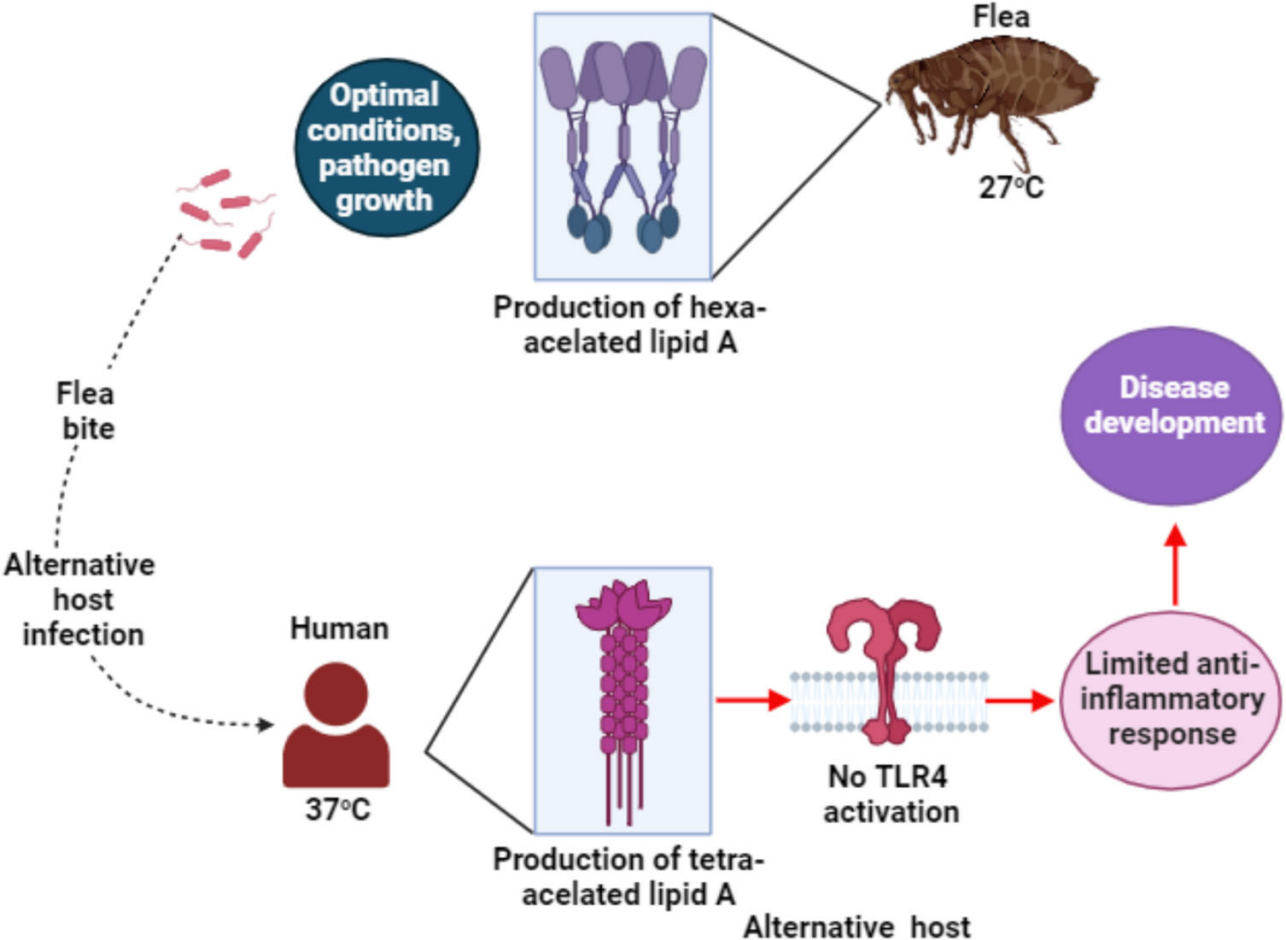
Schematic representation of the life cycle of a flea infected with *Yersinia pestis*. (Created with BioRender)

It is the carrier of the bubonic plague and murine typhus, which are spread by the eggs of fleas from one generation to the next (Farhang-Azad et al. 1985) (Figs. 7, 8). During World War 2, Japan employed these fleas as a biological weapon against the Chinese on a large scale. Lt. General *Shirō Ishii* approved his program (Frith 2012). Chinese deaths from the plague, which killed 500 000 people, were caused by fleas carried by low-flying planes that landed in Changed (Novick and Marr 2003; Lockwood 2008). Once *Yersinia pestis* reaches the flea digestive tract (27 °C), it produces a hexa-acylated form of lipid A and begins to proliferate in these favorable conditions. By biting a new host, the flea can further spread the disease. Once humans are accidentally bitten, the bacterium produces the tetra-acylated form of lipid A again to adapt to the human body temperature. However, the human TLR4 does not recognize this lipid A variant, and therefore, it is not able to activate the downstream signaling cascade. As a result, the immune response is ineffective as it must rely only on other defense mechanisms. *Yersinia pestis* invades the body undisturbed, causing a disease called plague.

#### Diptera

##### House fly

The house fly is a deadly biological weapon. It is a carrier of up to 100 distinct pathogen species, which can lead to illnesses such as parasitic worms, cholera, typhoid, salmonellosis, anthrax, TB, ophthalmia*,* and bacillary dysentery*.* Additionally, it is a significant vector for several viruses, including enteroviruses, poliomyelitis, and viral hepatitis (Förster et al. 2009). Additionally, several house fly strains are resistant to a variety of widely used pesticides (Georghiou and Hawley 1971; Keiding 1975). During WWII, as an entomological weapon against the Chinese, the Japanese dispersed disease by using flies afflicted with cholera.

##### Black flies

Black flies are little, robust flies, Sized between 3- and 6-mm narrow wings and a humped thorax. They are also referred to as buffalo gnats and turkey gnats. They drink birds’ and cattle’s blood. In the event of a mass fly attack, the animal dies. Acute toxemia frequently results in death. Low fly populations even have the effect of decreasing productivity. Additionally, they are vectors for filarial nematodes (*Onchocerca*), which cause bovine onchocerciasis, and protozoans (*Leucocytozoon*)*,* which cause leucocytozoonosis in poultry (Williams 2009). Black flies are parasitic nematodes that carry the *Onchocerca volvulus* parasite, which causes onchocerciasis in humans. The parasite, which can be used to spread disease to people, animals, and birds as a biological weapon, resides in human skin and is spread by flies taking food during feeding (Adler and McCreadie 2019).

##### The warble fly

These are big flies that parasitize cattle. They go by other names such as heel flies, bomb flies, and gadflies. They refer to their larvae as cattle grub or wolves. Certain species’ larvae also penetrate human tissues. Large, hairy, and range in color from orange to yellow, they resemble bees. Adults possess vestiges of their mouths and are free-living (Piper 2007). Paralysis results from tissue-invading cattle grub larvae entering the esophagus and spinal cord (Williams 2009). In humans, larvae of the *Hypoderma bovis* species create a condition known as intracerebral myiasis, which is characterized by an invasion of intracerebral tissues and symptoms like convulsions and intracerebral hemorrhage (Kalelioğlu et al. 1989). Cattle grub, or the larvae of warble flies, can be utilized as a biological weapon. Only in 1976, cattle grub losses were estimated to have cost US $360 million. *Hypoderma tarandi* is the caribou parasite that causes human eye myiasis, which results in glaucoma, uveitis, and retinal damage (Lagacé-Wiens et al. 2008). Therefore, warble flies can be used as a biological weapon against livestock if they are produced on a large scale in a laboratory.

##### Screw worm fly

Another biological weapon that can be used against livestock animals is the screw worm fly. The larvae of this parasitic fly feed on open wounds on warm-blooded animals (Alexander 2006). Only the host’s living, healthy tissues are attacked by their larvae. Cattle and other livestock animals are severely harmed by it (Williams 2009). Screw worms can result in tissue loss, the destruction of essential organs, and in severe situations, even death (California Department of Food and Agriculture 2022). During her life cycle, when looking for a host, a female screw worm can deposit up to 3000 eggs and travel up to 200 km (James 1947).

##### Midges that bite

These flies, range in length from 1 to 4 mm. Punks, sand flies, and no-see-ums are some other names for them (Whelan 2003). They bite with viciousness and persistence (Williams 2009). Arboviruses and various non-viral pathogens are transmitted by them (Linley 1985; Carpenter et al. 2013). They are virus vectors responsible for sheep and cattle developing blue tongues. They are also the source of epizootic hemorrhagic disease in cows. Horse onchocerciasis and blood protozoans are transmitted by poultry (Williams 2009). Hence, the disease can be spread among cattle and poultry using artificially infected biting midge colonies (Mullen and Durden 2009).

##### Mosquitoes

To infect humans, animals, and birds with disease, mosquitoes can also be employed as biological weapons. They serve as the carriers of various parasites and viruses. *Aedes aegypti* is the primary cause of viral diseases like yellow fever, dengue fever, and chikungunya (World Health Organization 2009). Similar to this, *Plasmodium* is the protozoan that causes malaria (Worrall et al. 2005). In order to attack the Soviet Union, the USA established a laboratory during the Cold War that could produce 100 million mosquitoes infected with yellow fever (Lockwood 2008). During Operation Big Buzz in 1955, 300 000 yellow fever-infected mosquitoes were dropped by the United States to test their survivability (Howie-Willis 2016).

#### Lepidoptera

The most common agricultural pest is Lepidoptera. *Malacosoma disstria,* a univoltine early-spring defoliator belonging to the Lasiocampidae family of Lepidoptera*,* is the culprit behind the forest tent caterpillar (FTC) outbreaks in North America (Parry 1995). The trembling aspen (*Populus tremuloides,* Salicaceae) is the main host tree species for fungal thermo toxicity (FTC) in the Canadian boreal forest. Since the 1940s, there have been documented cyclical outbreaks of FTC, which last one to four years (Romano et al. 2019) and happen roughly every ten years (Cooke and Lorenzetti 2006). FTC moths lay large clusters of eggs in the middle to end of summer. The eggs overwinter and hatch the following spring, just in time for the bud break of the host tree (Parry 1995). FTC is a gregarious feeder that maintains cohesion in colonies of up to several hundred individuals (Despland 2013).

#### Potential applications of honey bee weaponry in non-lethal crowd control strategies

Apart from the advantages mentioned above of beekeeping, honey bees can also be used for charitable purposes to control violent crowds because honey bees have a natural stinger that causes horrifying pain in people. Even though a honey bee’s sting is not dangerous to humans and can even boost our defenses against various illnesses, most people become anxious when honey bees are around. The mere sound of honey bees buzzing is sufficient to incite violent panic among the populace. The handling of a crowd of people protesting a government policy or action is a general issue for the federal government, state governments, and police in general. Therefore, the Indian army, provincial police, paramilitary forces, security guards, or police are employed to control the human crowd or mob at some event. Materials like fences, crowd control barriers, and stanchions are also being used. When there is a crowd, military personnel use less lethal weapons like batons to scatter the people. Sometimes, armored combat vehicles, water cannons, long-range acoustic devices, and police dogs, also referred to as sniffer dogs are employed. However, deadly weapons like pellet guns are also used against the crowd if they become difficult to control. Special weapons like rubber and wooden batons, an Indian lathi, a heavy leather or plastic whip, and vehicle-mounted water cannons are used to quell riots and angry crowds. The equipment mentioned above, which is used to control crowds, costs a significant amount of money and labor. Officers conducting crowd or riot control frequently wear protective helmets and carry shields and fences with them, which again cost a considerable amount of money and often cause issues for the officers as a means of defending themselves against the enraged mob.

Therefore, honey bees can also be utilized as an alternative method to control the unruly crowd and avoid this issue. As killing or harming people is not our primary goal, we want to scatter them. Although bee stings are terrifying for everyone, they are not dangerous unless a person has an allergy or is stung by many bees. Honey bees are, therefore, a safe way to control unruly crowds. Using tear gas can result in permanent harm, such as skull fractures, blindness, and even death (Clarot et al. 2003). Using pepper spray as a crowd control tool can cause short-term irritation and damage the stomach lining, bronchial airways, and eyes. Lack of enough outlets or water discharge points, or nozzles, is a limitation when using water cannons as crowd control devices, with water scarcity being the main one. In addition, a high-pressure pump with a limited effective range of 50–90 m is needed to discharge water. Those within the range of long-range acoustic devices suffer permanent hearing damage due to the loud sound it produces during crowd control. There won’t be any issues like this when employing honey bees. In general, police and customs officers rely heavily on sniffer dogs trained dogs that function as sensitive biological devices to locate hidden explosive devices and illegal drugs. Retraining dogs to identify a more limited range of drugs can be challenging, and training a new dog takes time. It is now known that honey bees can be utilized instead of sniffer dogs to detect explosive bombs and illegal drugs through their sense of smell. These days, honey bees are a beneficial tool for our military and paramilitary forces to save time when searching for explosive bombs and illegal drugs. About 10 lakh people are employed by seven paramilitary groups in India, and the Ministry of Home Affairs (MHA) is in charge of 46 battalions of Assam Rifles. These groups carry out a variety of tasks, such as providing internal security under army command by conducting counterinsurgency and border security operations, aiding the civil authority in times of need, and supplying communications, healthcare, and education in remote areas. They can also be used as a fighting force during wartime to protect nearby areas if necessary. Members of the team have enough time to themselves when there isn’t enough work for them, as special army and paramilitary forces live or stay outside the city. They don’t have any trouble raising honey bees because they live outside the city or in a forest. Since raising honey bees is an exciting hobby, it can be introduced to every office and camp for beekeeping. Since the raising of honey bee colonies and the production of honey are closely related, honey production will provide our forces with a vital source of nutrition. These workers receive low pay, which leads to malnutrition in their offspring. If beekeeping succeeds, they will be able to obtain honey directly, which is nutrient-rich, providing them and their kids with the best food possible and helping them to overcome the issue of malnutrition. They can also make money by selling honey and other bee products like bee wax on the local market. The company’s head may form different teams in terms of the team. Designating at least ten campus personnel to care for and oversee the honey bee colonies is possible. They can be taught the scientific and contemporary methods of beekeeping, rearing, and preserving the stock culture of honey bees. The honey bee stock team will only be called upon in the event of a demonstration or other uncontrollable circumstances. Training about 20 people to release the bee packages at the protest location is possible. Each team member will wear a netted helmet over their head in addition to the standard official protesting device worn over their body. Following a thirty-minute announcement, each member with a honey bee package containing ten to twenty thousand bees will appear and release their honey bee packet. Following their release, honey bees fly and hover over the crowd, which causes people to become fearful of them and quickly scatter. Given the economics of beekeeping, at least three lakhs are needed to raise 100 bee hives, and an additional 1 lakh is needed for maintenance. Thus, to raise at least 100 bee hives, a total of 4 lakhs is needed. There are almost fifty thousand bees in a single hive. Thus, roughly 50 000 000 bees reside in 100 bee hives. A 500-g cluster of honey bees contains roughly 4500–5000 bees, as a single bee weighs between 0.1 and 0.12 g. Therefore, 1000 clusters of roughly 500 g can be produced on average from a single military campus. Honey production will undoubtedly rise if beekeeping is encouraged in two or three locations throughout each district, as this will enhance the pollination of various crops, particularly fruit crops (Lockwood 2008). Even though India gained independence from the British in 1947, many issues still plague the country, the most significant of which are poverty, unemployment, and terrorism. To use honey bees for crowd control, we must first engage in beekeeping. By doing so, we can prevent issues with unemployment and poverty because beekeeping produces much more than just honey. Terrorism is currently one of India’s biggest issues. Terrorist attacks have become more frequent in India in the past year. The public’s security has been put at risk by this. Therefore, the government invests significant money in bolstering the military force each year to combat terrorist activity. Even so, there is some degree of control over these activities. Since bees become more virulent when their hives are destroyed, we can manage the problem of terrorism by encouraging bees to stay in hilly or bordering areas. Early humans’ employment of bees was most likely the first instance of entomological warfare. The bees or their nests were thrown into caves to drive the enemy into the open. Ancient Romans and Heptakometes used honey bees and their product, honey, for personal and national defense (Lockwood 2008).

#### Ticks

Members of the Arachnida class include ticks, which belong to three families, two of which are known to carry diseases that can infect humans. There are thirteen genera in the family Ixodidae, of which *Amblyomma*, *Dermacentor*, and *Ixodes* are the ones that infect people in the USA (de la Fuente 2023). There are five genera in the Argasidae family; in the USA, People can contract diseases only from *Ornithodoros* (Sarwar 2017).

Lyme disease is the most common tick-borne illness in the USA (Walker 1998). Human granulocytic ehrlichiosis (HGE) is caused by the *Ehrlichia* species tentatively named *Ehrlichia phagocytephila* (*Ehrlichia equi*), as well as *Ehrlichia wingii* and *Ehrlichia chaffeensis* (Walker 1998). The bacterium *Rickettsia rickettsii* is responsible for Rocky Mountain spotted fever. This small, pleomorphic, obligatory intracellular parasite infects blood vessel smooth muscle and endothelial cells (Spach et al. 1993).

## Biodefense and protection from a biological weapon

Public education and awareness of the risks and hazards associated with biological agents are crucial. Food that has been cooked, boiled, chlorinated, or filtered, drinking water and controlling pests and rodents interventions must start immediately; it is essential to include clinical isolation of both suspected and confirmed cases. An early correct diagnosis is crucial in managing victims of biological assault. Thus, a system of specialized Establishing laboratories is necessary for a laboratory confirmatory diagnosis. The current system for monitoring diseases, mass vaccination campaigns, vector control measures, and programs in the suspected area must be further investigated assiduously. Improving the expertise and abilities of clinicians is crucial in managing the damaging attack’s effect. Given bioterrorism and associated infections, they will continue to be uncommon and imaginative (Centers for Disease Control and Prevention 2013). Many BW threat agents have effects that can be avoided or lessened with proper safety measures including vaccinations, treatments, and prophylactic measures both before and after exposure, and there are options for protective apparel to offer a defense. Employees need to have received all necessary vaccinations. Employment poses a risk before going into a region of operation where a BW agent is present. If an attack appears likely or is documented as having happened, command directed for every employee, chemoprophylaxis would be appropriate in the vicinity. It is wasteful and inefficient to put everyone in the area of a possible target on extended, regular use of antibiotic prophylaxis in the absence of such a condition of threat (Centers for Disease Control and Prevention 1999). Before troops are sent into action, all vaccinations should be given in a timeframe that allows the initial protection to take effect. Troops cannot be deployed to the theater of operations until they receive their vaccinations, which must be administered as soon as the mission permits. Certain vaccinations are administered in addition to either post-exposure or pre-exposure chemoprophylaxis as a defense. In the operating area, vaccinations, medications, and chemoprophylaxis are necessary. Using protective gear in addition to chemoprophylaxis for BW agents for whom a specific vaccine is not available is possibly employed to provide security. Vaccination is a crucial and effective way to offer ongoing defense against BW threats before and during hostile actions. According to CDC, immunizations against biological weapons agents are limited in number. Several vaccines have been developed to protect laboratory workers and individuals in areas where these diseases are common (Centers for Disease Control and Prevention 2013).

## Final thoughts and suggestions

Throughout human history, the use of biological weapons has been rare, and it is still determined whether they are genuinely effective as combat tools. The high risk that natural infections pose to human health, such as influenza virus infections, where a sizable population is affected, often undervalues their potential use as biological weapons due to the ease of spread. Over the past century, interest in biological weapons has persisted, especially in contexts where defying or opposing regional hegemony may necessitate unorthodox weaponry. As a result, biological weapons continue to pose a significant risk. Zoonosis is the most likely infectious disease that bioterrorists will use to create BWs, so cooperation between veterinary and human medicine can be beneficial. It spreads when barriers between different animal species crumble the body of veterinary and human medicine knowledge. Combining surveillance for human and veterinary health and making an effort are crucial when combating bioterrorism. This endeavor will necessitate better teamwork and communication and a unified and well-coordinated effort by scientists, health veterinarians, epidemiologists, and healthcare professionals, which are required to reduce the impact of bioterrorism on a global scale. As a result, we advise that widespread public awareness be raised before, during, and following such an attack (presumably). Individuals ought to be informed about the possibility of a biological Weapon and the different actions necessary to examine our capacity for biodefense and ensure there are enough defenses against new dangers. The national administration ought to offer emergency medical coverage during an assault by bioterrorists.

## Conclusion and recommendation

Utilizing insects as biological weaponry is a highly affordable and successful tactic. They are easily employed to harm enemy cattle and crops and infect enemies with disease. The delivery methods and the choice of insect vectors can be influenced by climatic conditions. For example, the use of mosquitoes as vectors might be more prevalent in tropical climates, while the use of ticks might be considered in temperate regions. However, in contrast with conventional weapons, their sluggish action and illegality make their use in combat unlawful; hence, laws and regulations should be implemented to forbid their proliferation or use in combat. To stop biological weapons from falling into the hands of terrorists and being used to incite terrorism, there should also be checks and balances. Increased export and import security, as well as airport security, are necessary to prevent the smuggling of biological weapons across international boundaries. To this end, entomological experts should be part of anti-terrorism investigations, border patrol, and airport security teams. Due to their ability to reproduce themselves and their ability to induce delayed morbidity and death after exposure or infection, vector-borne illnesses pose a hazard today. Most pathogenic organisms, especially zoonosis that are employed as biological weapons can be introduced into a target population without running the risk of being discovered right away. This article exemplifies how an enemy could target US military troops and civilians by using insects and other arthropods. Some suggestions were made on how to lessen the threats mentioned (Paederus beetles, Medflies, Rift Valley fever). Still, all military personnel may diminish the risks of vector-borne diseases by doing straightforward things. When terrorism is engaged, the threat posed by invasive pests and the diseases they may transmit is increased. The essential framework required to address the entomological threats we face is provided by research conducted by academic institutions, numerous federal agencies (such as USDA-ARS), the US military (such as USAMRIID), and organizations capable of coordinating emergency response activities (such as DHS, Federal Emergency Management Agency, state and local authorities). A comprehensive response to entomological terrorism would be desirable, but this may not be achievable given the associated financial and logistical difficulties. As with any terrorism, the most crucial defense against entomological terrorism is constant watchfulness on the part of the military, government organizations, people, and allies. Future research should consider the regional differences to provide a more nuanced understanding of the historical and potential use of entomological warfare, specifically the impact of climate change on the distribution of insect vectors and the potential implications for the spread of insect-borne diseases in the context of biological warfare. Also, the development of effective countermeasures and robust defense strategies must be tailored to the specific regional contexts in which entomological warfare might be employed, addressing the unique challenges presented by varying climates, ecological conditions, and potential vectors. Furthermore, it is imperative to delve into the ethical and legal considerations surrounding the use of entomological warfare across different regions, with careful attention to cultural norms, environmental impacts, and geopolitical factors that could influence both the likelihood of its use and the response to it.

## Abbreviations

ARS: The Agricultural Research Service
ATA: Alimentary toxic aleukia
BW: Bioweapon
CDC: Centers for Disease Control and Prevention
DHS: The Demographic and Health Surveys
DRDE: Defense Research and Development Establishment
FAO: Food and Agriculture Organization
FTC: Forest tent caterpillar
HGE: Ehrlichiosis granulocytic in humans
HME: Human monocytic ehrlichiosis
IAEA: International Atomic Energy Agency
Lt. Gen: Lieutenant General
MHA: Ministry of Home Affairs
RAMP: Rapid measurement platform
SEB: Staphylococal entero toxin (type B)
UAVs: Unmanned aerial vehicles
USDA: United States Department of Agriculture
USSR: Union of Soviet Socialist Republics
USAMRIID: US Army Medical Research Institute of Infectious Diseases
VEE: Venezuelan Equine Encephalitis
WHO: World Health Organization
WWII: World War II
